# Exploring the mechanism of Dahuang-Tusizi drug pair in the treatment of diabetes nephropathy based on network pharmacology and immune infiltration analysis

**DOI:** 10.1097/MD.0000000000036020

**Published:** 2023-11-24

**Authors:** Wenjing Liu, Ling Yuan, Mengying Che, Shaozhang Hou, Fandi Meng, Duojie Xu, Yi Nan

**Affiliations:** a Key Laboratory of Ningxia Minority Medicine Modernization Ministry of Education, Ningxia Medical University, Yinchuan, China; b College of Pharmacy, Ningxia Medical University, Yinchuan, China; c Traditional Chinese Medicine College, Ningxia Medical University, Yinchuan, China.

**Keywords:** Dahuang-Tusizi drug pair, diabetes nephropathy, immune infiltration, molecular docking, network pharmacology

## Abstract

The study aimed to explore the key targets and molecular mechanisms of Dahuang-Tusizi drug pair (DTDP) in the treatment of diabetes nephropathy (DN) based on the GEO database by using network pharmacology combined with molecular docking and immune infiltration. The active components of the DTDP were screened using the Traditional Chinese Medicine Systems Pharmacology database and the Swiss Target Prediction database. The differential genes of DN were retrieved from GEO databases. Next, the intersecting targets of drug and disease were imported into the String database for protein–protein interactions network analysis, and the core targets were identified through topological analysis. Gene Ontology analysis and Kyoto Encyclopedia of Genes and Genomes enrichment analyses were performed with the help of the Metascape database and gene set enrichment analysis database. Subsequently, molecular docking was performed to verify the binding activity of the key component and the key target. The Nephroseq V5 database was used to verify the clinical relevance of DN and core genes. Finally, the Using CIBERSORT Algorithm to analyze the immune Infiltration of DN Gene Chip. The network analysis showed that 25 active ingredients of DTDP were associated with 22 targets in DN. The key active ingredients (Sesamin, quercetin, EUPATIN, matrine, beta-sitosterol, isorhamnetin, etc.) and the core targets (JUN, EGF, CD44, FOS, KDR, CCL2, PTGS2, and MMP2) were further identified. Enrichment analysis revealed signaling pathways including TNF, MAPK, and IL-17 signaling pathway. Molecular docking results showed that there was a strong affinity between the key components and core targets. The results of immune infiltration found that the proportion of macrophages in DN tissues was significantly increased. Our findings demonstrated that the characteristics of DTDP in treating DN are “multiple components, multiple targets and multiple pathways.” We predicted that DTDP may inhibit inflammation related pathways by regulating key genes, reducing macrophage infiltration. Thus, inhibiting inflammatory response to reduce glomerular damage and delay the development of DN.

## 1. Introduction

Diabetic nephropathy (DN) is a common and serious microvascular complication of diabetes mellitus (DM). According to epidemiological studies, more than 425 million people worldwide are currently affected by diabetes. If no action is taken, more and more people with diabetes worldwide will rise to 629 million by 2045.^[1]^ As the prevalence of diabetes has increased, the incidence of DN has increased rapidly. Being expected to 30% to 40% of diabetic patients develop DN, finally, the one third of DN patients develop end-stage renal disease. Currently, it has been shown that the mechanism of DN is the result of a combination of factors such as glucose metabolism disorders, changes in glomerular hemodynamics, inflammatory reactions, cytokines, endothelial damage, oxidative stress, ischemia and hypoxia, and genetic susceptibility. Chronic renal hypoxia, caused by various factors such as hyperglycemia, endothelial damage, and inflammatory responses, is one of the main causes of the occurrence and development of DN. Hypoxia is considered as an etiological factor in the progression of renal injury. HIF1α is a major transcription factor that transduces an array of cellular processes to let the cells adapt to hypoxic injury. Therefore, a sustained increase in HIF1α is a major adaptive stimulus to the hypoxic conditions.^[2]^ By regulating its target ZEB2 gene (Zinc finger E-box-binding homeobox 2), it affects pathological and physiological processes such as glucose, energy metabolism, inflammation, and plays an essential role in the occurrence and development of DN.^[3–5]^ There is a growing body of evidence that hypoxia can cause kidney damage, inflammatory reactions, loss of podocytes and abnormal function, and reduced glomerular filtration rates leading to proteinuria. If not intervened in a timely manner at an early stage, this is a huge financial burden on society.^[6]^ Western medications are used to treat DN by regulating blood glucose, blood lipid levels and blood pressure. Early medical studies have shown that angiotensin-converting enzyme inhibitors and angiotensin receptor blockers (angiotensin II receptor blockers) as first-line treatment for DN can reduce proteinuria, The role of delaying the progress of renal dysfunction.^[7]^ However, the side effects of angiotensin-converting enzyme inhibitors/angiotensin receptor blockers limit the use of these drugs. Therefore, the search for safe and effective therapies for DN is of great urgency.

Nowadays, a number of evidence shows that traditional Chinese medicine (TCM) and herbal medicines have been playing an essential role in the treatment of DM and its complications. TCM is clinically proven effective and focuses on the functioning of the coordinated system.^[8]^ Numerous experts pay considerable attention to exploring the Chinese medicine compound treatment of DN.^[9–11]^ Dahuang-Tusizi drug pair (DTDP) consists of Dahuang and Tu-Si-Zi. As we all know, Dahuang (Rhubarb) is used as a cathartic in Chinese medicine.^[12]^ In modern pharmacology, Dahuang has long been used in China as an anti-bacterial, anti-inflammatory, anti-fibrotic and anti-cancer medicine.^[13,14]^ It contains a variety of active ingredient, including anthraquinone, anthrone, acetophenone, flavonoids, and polysaccharides.^[15–17]^ A research study indicated that Dahuang has a significantly inhibitory effect on the elevated metabolism of kidney, which effectively prevents the accumulation of extracellular matrix and promotes its effective degradation, modulates inflammation, delaying sclerosis of the kidney.^[18]^ Tu-Si-Zi (Semen Cuscutae) is a commonly used as tonifying the kidney in traditional Chinese herbal drug, prepared from the seeds of Cuscuta chinensis Lam,^[19,20]^ which exhibits the anti-inflammatory,^[21]^ antioxidant,^[22]^ and anti-cancerous properties.^[23]^ Through clinical research, Tu-Si-Zi performed a bright future in the therapy of DM and its complications.^[24]^ In experimental study, it has been shown to improve kidney function, regulate the body’s endocrine and immune system.^[25]^ The chemical constituents of Tu-Si-Zi have been more comprehensive investigated, Its main components are flavonols such as quercetin, kaempferol, and sesamin, etc.^[26]^ These compounds may be responsible for the biological activity of the drug. The above research suggested that DTDP has a potential role in treating DN. Thus, the tonic of Tu-Si-Zi and laxatives of Dahuang were used together when treating renal disorders in traditional Chinese medicine.

**Figure 1.**
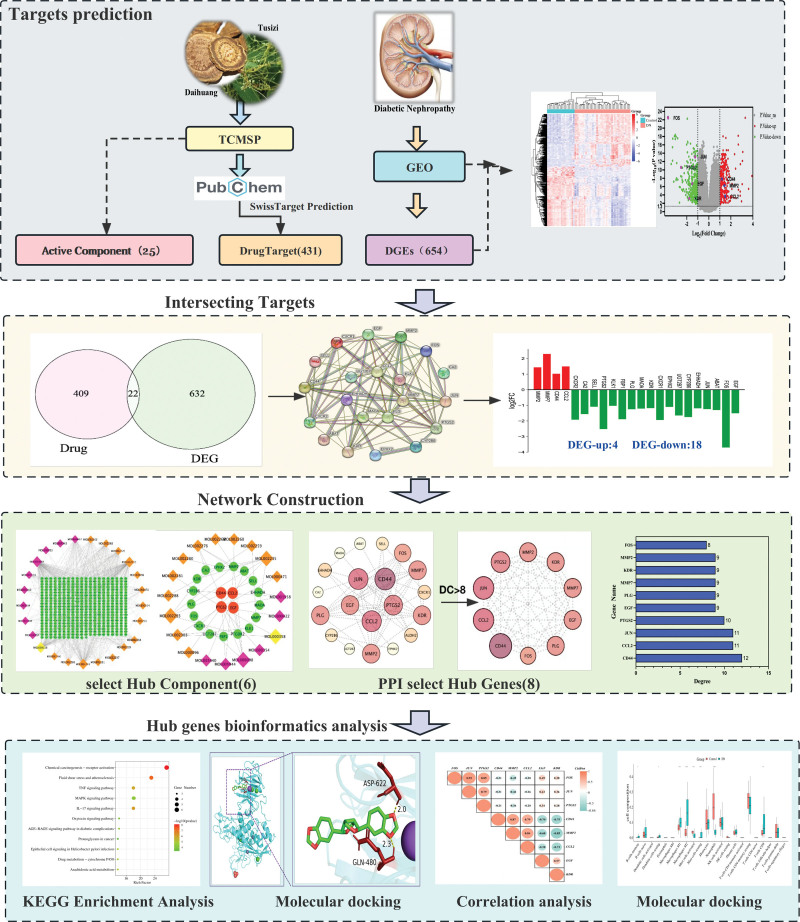
Flowchart of the current research study.

Network pharmacology is an interactive drug-target-gene-disease based network that includes chemoinformatics, bioinformatics, network biology, and pharmacology.^[11,27]^ It is employed to elucidate the interplay between the components, targets and pathways of the active ingredients of TCM,^[28]^ and it has been developed into a comprehensive tool to uncovered bioactive constituents and fundamental mechanisms of TCM from a holistic standpoint.^[29]^ Therefore, in this study, we investigated the assembly of “multi-component, multi-target” network of TCM compounds through network pharmacology and bioinformatics analysis. It aim to predict, reveal and clarify the molecular mechanism of DTDP in treating DN, hoping to provide more advice and guidance for the treatment of DN. The flowchart is shown in Figure 1.

## 2. Materials and methods

### 2.1. GEO gene chip data collection and annotation

We retrieved the GEO database (https://www.ncbi.nlm.nih.gov/geo/) using keywords such as “T2D diabetic nephropathy” and “glomeruli.” The probes are converted to the corresponding gene symbols based on the GPL17586 platform annotation information. With the help of the GEO2R software, the above chips are grouped, normalized and further processed. The screening conditions of differential genes were set as | log2FC |>1 and *P* < .05, and the significantly expressed differential genes (DEGs) were obtained. The differential gene heat map was drawn online by using bioinformatics platform (http://www.bioinformatics.com.cn/), and the original gene volcano map of the chip was drawn by using the mapping software GraphPad Prime 8.

### 2.2. Acquisition of chemical components and targets for DTDP

The active components of DTDP were downloaded from the Traditional Chinese Medicine Systems Pharmacology database (https://tcmsp-e.com/). Assessing the efficacy of drugs administered orally into the systemic circulation is greatly reliant on oral bioavailability (OB), which stands as a crucial pharmacokinetic characteristic. Drug evaluates the potential of a compound to be a drug. Only molecules with increased OB and drug like (DL) can exhibit excellent pharmacological activity. We limited conditions of OB ≥ 30% and DL property ≥ 0.18; the 2D structures of active ingredients were retrieved through PubChem (https://pubchem.ncbi.nlm.nih.gov/), and their SDF files were downloaded. Then import the SDF file into Swiss Target Prediction (http://www.swistargetprediction.ch/) predicts potential drug targets. subsequently, the active ingredient and corresponding target of Cuscuta chinensis were imported into Cytoscape 3.8.0 software and the component target network was constructed.

### 2.3. Intersection of disease genes and drug genes

First, take intersection between DTDP and DN chip differential gene, and Venn diagram was drawn. The predicted target genes of DTDP active components were intersected with up-regulated genes and down-regulated genes in differential genes of DN, respectively. Draw a Venn diagram in R using the Venn diagram package.

### 2.4. PPI analysis of protein–protein interactions

The intersection of target genes were introduced into the STRING database (https://string-db.org/) to construct the protein–protein interactions (PPI) network. The biological quality control is facilitated by the STRING database, which consolidates numerous networks of protein-protein associations. For the selection of the PPI target, we prioritize those with a confidence score exceeding 0.7 and other parameters remain default. The PPI network was screened by using Cytoscope and each node was scored using the plug-in CytoNCA. The Core networks were created and visualized using Cytoscape. The core targets in core network were scored according to the degree centrality (DC).

### 2.5. Gene Ontology and Kyoto Encyclopedia of Genes and Genomes enrichment analysis

The intersection target of DEGs and DTDP was subjected to an analysis using the Metascape database (https://metascape.org/) along with Gene Ontology (GO) analysis and Kyoto Encyclopedia of Genes and Genomes (KEGG) analysis. selected the filter condition organization as “hsa,” set Min Overlap = 3, *P* value Cutoff < .05, Min Enrichment = 1.5. The top 10 enrichment terms of biological process (BP), cellular component (CC), molecular function (MF), and KEGG enrichment analysis were presented in a bubble diagram using the bioinformatics platform. By utilizing the Cytoscape software, the KEGG relational network is established through the incorporation of either the Pathway ID number or the Pathway enriched genes. Next, an assessment of the network entails the computation of the neighboring nodes’ quantity, followed by the evaluation of the node size within the network, which is contingent upon the aforementioned neighboring node count.

### 2.6. Gene set enrichment analysis enrichment analysis

Gene set enrichment analysis (GSEA) is a scientific technique used to analyze microarray data concerning genome-wide expression profiling. This method allows for the identification and sequencing of genes based on variations in their expression levels between 2 samples. In this study, GO and KEGG analyzes were performed on all detected genes using GSEA software (version 3.0). Unlike the GO and KEGG analyses, the GSEA enrichment analysis does not necessitate a differential gene threshold. It effectively assesses if a group of genes is distributed randomly within a specific phenotype or is subject to abnormal regulation, thereby ascertaining the biological significance. Consequently, we utilized GSEA to explore the variances in biology between samples of DN and regular kidney tissues.

### 2.7. Molecular docking

To obtain the 2D structure of the key components of DTDP, we imported the key active ingredients into PubChem and converted the 2D structure to 3D using chem3D software. Meanwhile, we used the PDB (https://www.rcsb.org/) database to obtain the core target protein PDB format. Pymol software was used to remove water molecules and short-molecule ligands of the proteins. The docking process combined the AutoDock tool with the currently widely used molecular docking strategy-AutoDock Vina, and improved the speed and accuracy of molecular docking through various evaluation functions. The edited results were done by Vina. Binding of drug to targets can be visualized using heatmaps. Using the Pymol molecular alignment visualization software, our findings indicate that the lower the binding energy, the better the alignment.

### 2.8. Nephroseq v5 validation

The Nephroseq V5 online database (http://v5.nephroseq.org) is an extensive noncommercial platform for exploring and analyzing gene expression data related to kidney disease. To ensure the reliability of our findings, we assessed the expression levels of essential genes linked to DN in comparison to those of normal controls, employing Nephroseq V5 database. We considered a *P* value lower than .05 as indicative of statistical significance.

### 2.9. Expression of core genes in kidney single cell dataset

Online database for analysis of renal single-cell datasets: the Kidney Interactive Tranomics database (http://humphreyslab.com/SingleCell/). The core genes were first searched by entering each of the 8 core genes in the home page of the Kidney Interactive Tranomics database to analyze the expression of the core genes in a cluster of single kidney cells. The output results in 3 images, which are the single-cell clustered t-SNE map of the dataset, the t-SNE map of the core genes, and the core gene expression violin map for each cell class group.

### 2.10. Receiver operating characteristic curve of core genes

ROC (receiver operating characteristic) analysis for diagnostic biomarkers is a common method for evaluating diagnostic accuracy, characterized by the combination of sensitivity and specificity. In medical research, ROC curves have been used to assess the diagnostic efficacy of core genes, to study the predictive accuracy of core genes for diseases, and to determine cutoff values. The corresponding sensitivity and specificity are calculated at different boundary points in a continuous variable, with sensitivity in the vertical coordinate and 1 − specificity in the horizontal coordinate, as indicated by the larger area and better sensitivity under the ROC curve. Plotting the ROC curves of core genes versus DN was carried out through the microbiology letter (http://www.bioinformatics.com.cn/keywords=ROC), and the diagnostic value of the core genes was assessed through the AUC values.

### 2.11. Immune infiltration analysis

The CIBERSORT algorithm in R was used to quantify the fraction of the 22 types of immune cells in the merged data set. To estimate the proportion of different immune cell types in glomerular tissues affected by DN and in healthy tissues, we employed deconvolution to process marker gene expression values. The immune cell types analyzed include M1 macrophages, M2 macrophages, plasma cells, static memory CD4^+^ T cells, γδ T cells, and mast cells, adding up to a total of 22 types. A plot illustrating the fractional composition of each immune cell type was generated for all samples. At the same time, correlation analyses were computed between hub genes and 22 immune cells and their correlations were visualized using a thermogram. According to the calculated correlation coefficient, the correlation strength between the pivotal genes and 22 immune cells was determined, and the “ggpubr” software package was used for visualization.

## 3. Results

### 3.1. GEO gene chip and differential gene analysis

**Figure 2.**
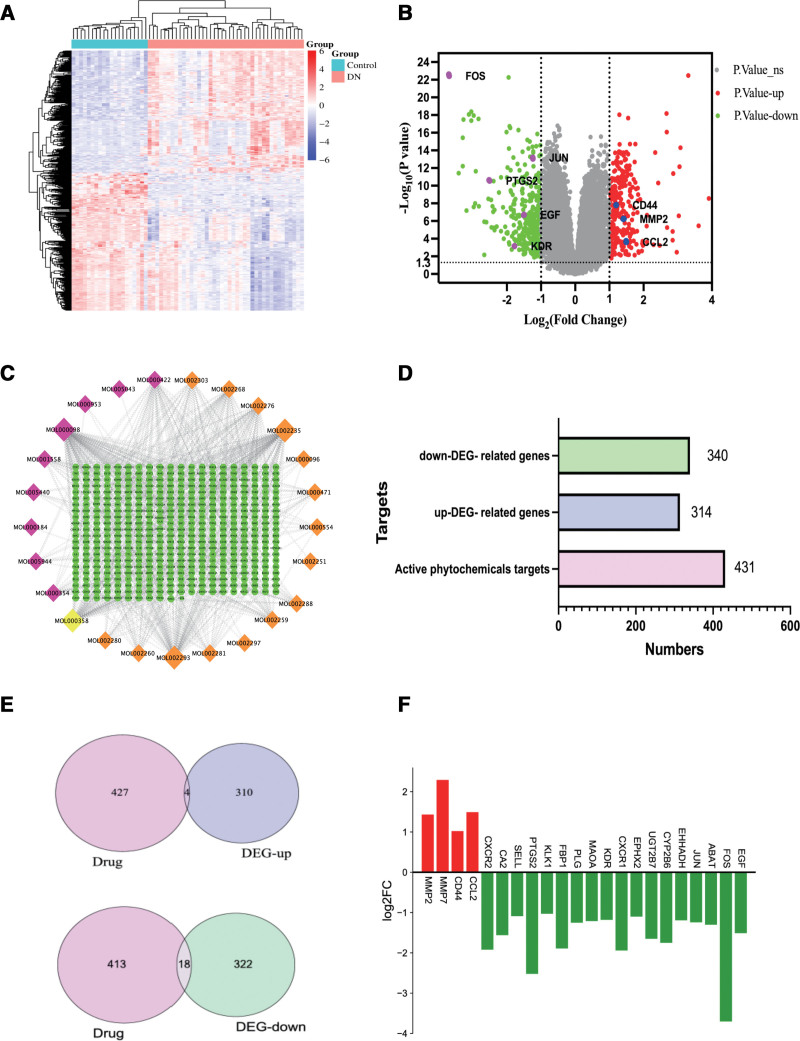
DEGs and drug target acquisition. (A) The heat map of DEGs. In the heat map, the horizontal axis represented samples (41 DN group and 20 normal control group), the vertical axis represented the DEGs, and the color represented the relative gene expression level, with red being elevated expression and green being low expression. (B) The volcano of DEGs. In the volcano diagram, the green part represents genes that are down-regulated in the normal group, the red part represents genes that are up-regulated in the DN group, and the gray parts represent genes that have no difference between the normal group and the DN group. (C) Network diagram of drug components and drug targets. There are 25 active phytochemical and 431target genes of DTDP (orange for Dahuang, pink for Tusizi, yellow for consensus components). (D) Bar graph of Potential protein targets of active phytochemicals in DTDP and down-regulated and up-regulated genes of DEGs. (E) Intersection of up-regulated and down-regulated genes of DEGs by DTDP drug targets respectively. (F) Bar graph of 22 down-regulated and up-regulated genes. We use the red and green respectively to demonstrate the up-regulated and down-regulated DEGs. DEGs = differential genes, DN = diabetes nephropathy, DTDP = Dahuang-Tusizi drug pair.

The dataset contains 61 samples (including 41 DN and 20 normal controls). the results showed the total of 32,658 genes were obtained by microarray analysis of the GSE96804 gene chip, and the DEGs screening conditions were set to |log2FC|>1 and *P* < .05. After screening, a total of 654 DEGs were screened from the GSE96804 dataset of T2D diabetic patients and healthy controls. DEGs results were used to draw a heat map and volcano diagram, as shown in Figure 2A and B, respectively.

### 3.2. Drug chemical components and targets

We obtained the active ingredients of DTDP with OB ≥ 30% and DL ≥ 0.18 as the screening cutoff point. A total of 25 active ingredients were obtained: 15 active ingredients from Dahuang and 11 active ingredients from Tusizi; while MOL000358 was consensus components. These active phytochemicals are given in Table 1 with their OB and DL. The 2D structure of the ingredients was imported into Swiss target prediction to predict the potential targets of the active ingredients. After combining and eliminating duplicates, a total of 431 target genes were identified for the aforementioned drugs. At the same time, 314 genes were up-regulated while 340 genes were down-regulated in the DEGs (Fig. 2C and D).

**Table 1:** These active phytochemicals of DTDP.

Herb name	ID	Phytochemical name	OB (%)	DL
Dahuang (Radix Rhei Et Rhizome)	MOL002235	EUPATIN	50.8	0.41
	MOL002251	Mutatochrome	48.64	0.61
	MOL002259	Physciondiglucoside	41.65	0.63
	MOL002260	Procyanidin B-5,3’-O-gallate	31.99	0.32
	MOL002268	rhein	47.07	0.28
	MOL002276	Sennoside E_qt	50.69	0.61
	MOL002280	Torachrysone-8-O-beta-D-(6’-oxayl)-glucoside	43.02	0.74
	MOL002281	Toralactone	46.46	0.24
	MOL002288	Emodin-1-O-beta-D-glucopyranoside	44.81	0.8
	MOL002293	Sennoside D_qt	61.06	0.61
	MOL002297	Daucosterol_qt	35.89	0.7
	MOL002303	palmidin A	32.45	0.65
	MOL000358	beta-sitosterol	36.91	0.75
	MOL000471	aloe-emodin	83.38	0.24
	MOL000554	gallic acid-3-O-(6’-O-galloyl)-glucoside	30.25	0.67
	MOL000096	(-)-catechin	49.68	0.24
Tusizi (Cuscutae Semen)	MOL001558	sesamin	56.55	0.83
	MOL000184	NSC63551	39.25	0.76
	MOL000354	isorhamnetin	49.6	0.31
	MOL000422	kaempferol	41.88	0.24
	MOL005043	campest-5-en-3beta-ol	37.58	0.71
	MOL005440	Isofucosterol	43.78	0.76
	MOL005944	matrine	63.77	0.25
	MOL000358	beta-sitosterol	36.91	0.75
	MOL000953	CLR	37.87	0.68
	MOL000098	quercetin	46.43	0.28

DL = drug like, DTDP = Dahuang-Tusizi drug pair, OB = oral bioavailability.

### 3.3. Intersection of DEGs and drug targets

A Venn diagram was constructed by intersecting the up-regulated and down-regulated genes in the DEGs with the targets of DTDP. Analysis of Figure 2E reveals that there were 22 intersection targets recognized between the DEGs and DTDP, with 4 genes being up-regulated and 18 genes being down-regulated. These 22 genes are visualized in the form of a heat map and bar graphs, as demonstrated in Figure 2F.

### 3.4. PPI network construction and recognition of hub genes

**Figure 3.**
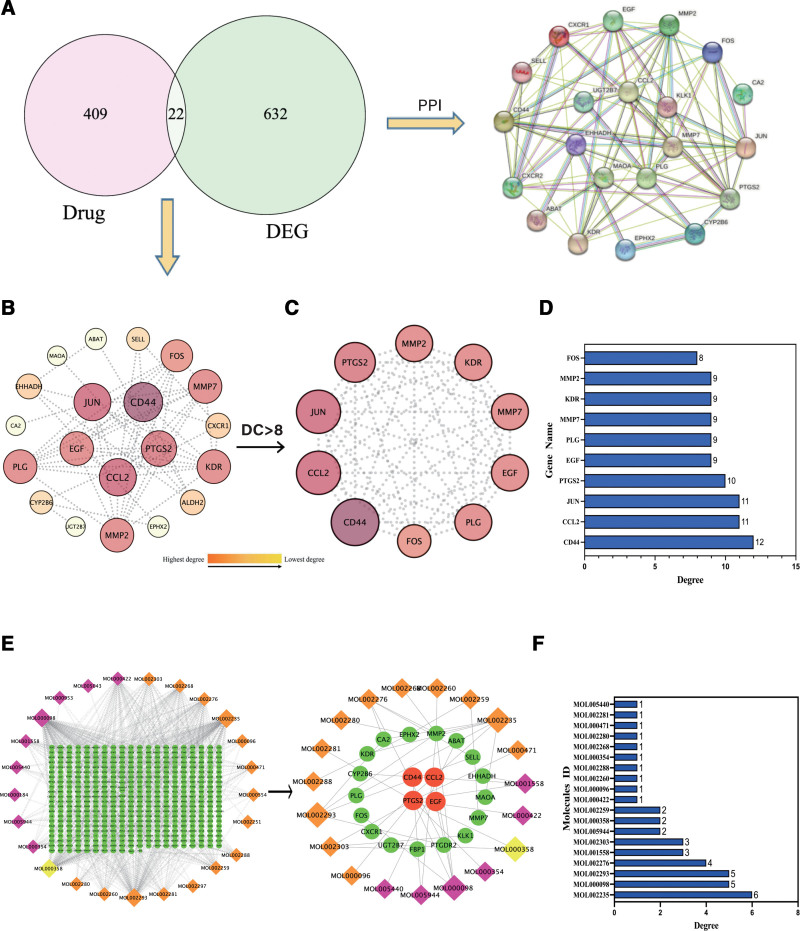
Screening core components and core objectives of DTDP in the treatment of DN. (A) Venn diagram of the intersection of the DTDP target and DN genes. PPI network was constructed with STRING. (B) The PPI network of the 22 intersecting targets was constructed using Cytoscope software. Each node’s color denotes the degree, from red (highest) to yellow (lowest), as the node degree decreases. (C) Screening the top 10 core genes in the network according to the degree. (D) the degree of top 10 core genes were drawn by Bar graph. (E) Screening core components. In the Cytoscape software, components corresponding to 22 interleaved targets are extracted from the component-target network of DTDP, and the PPI network screening graph was drawn based on the ranking of the degree values. (F) The histogram shows the degree value of the top 19 core components. DC = degree centrality, DN = diabetes nephropathy, DTDP = Dahuang-Tusizi drug pair, PPI= protein–protein interaction.

To conduct a thorough investigation of the 22 crossover genes, we utilized the String online database for PPI analysis (Fig. 3A). The interaction network is based on targets with a confidence level of 0.7. Visualization was performed using Cytoscape software after removing isolated nodes (MAOA and KLK1) and showing partially connected nodes. Figure 3B displayed the PPI network, comprising of 20 nodes and 61 edges. We assessed each node’s score using the CytoNCA plugin, screened the clusters within the PPI network, and identified the central gene based on the highest score. To select the central gene, we applied the following criteria: DC > 8. Consequently, Figure 3C depicted a total of 10 nodes and 44 connections. The core network was screened by degree score and contained 10 targets. Such as JUN, EGF, CD44, FOS, KDR, CCL2, PTGS2, MMP2 and additional genes formed the core of the network and the network diagram was obtained in Figure 3D.

### 3.5. Hub compound-target network analysis

We screened the key drug component networks corresponding to the core targets from the above networks. In addition, a hub compound-target network was constructed between 19 active phytochemicals and 22 potential anti-DN core targets, as shown in Figure 3E. Each of the 19 active phytochemicals was applied to 22 potential anti-DN core targets. The 19 active phytochemicals are shown in Figure 3F. There used to be a bar graph in the hub network based on their degrees. Six potential anti-DN core components: sesamin, quercetin, EUPATIN, sennosideE_qt, sennoside D_qt and palmidin A were selected for molecular docking studies.

### 3.6. GO and KEGG enrichment analysis

**Figure 4.**
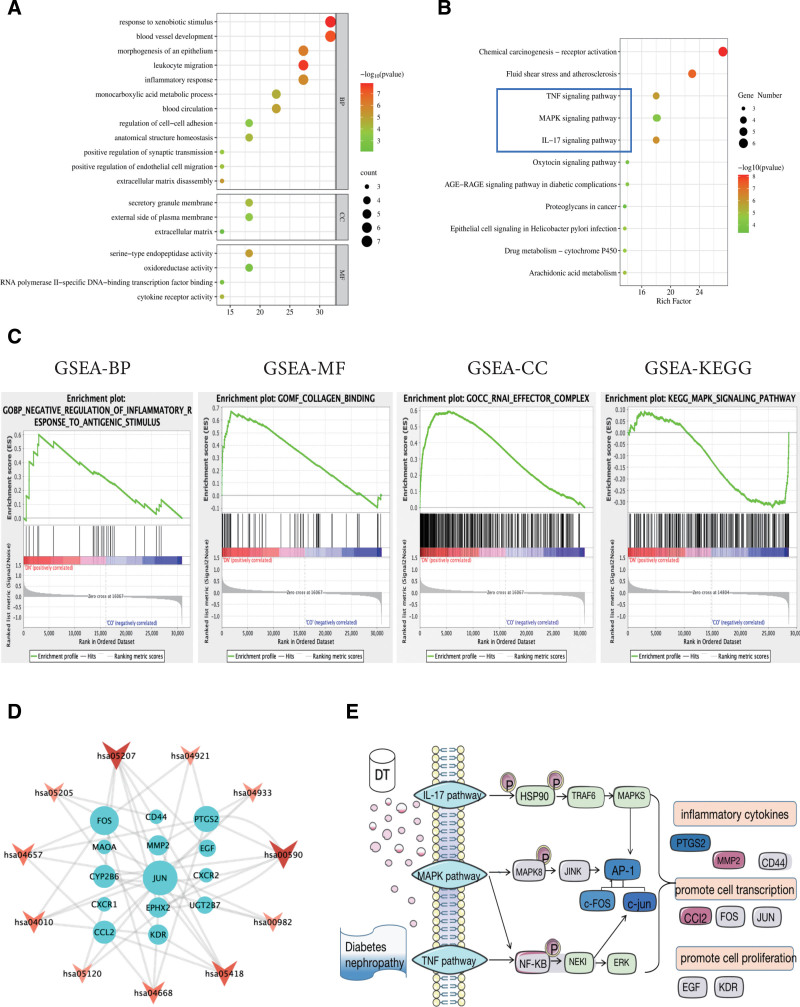
GO and KEGG enrichment analysis. (A) GO enriched analysis of DTDP treatment of DN, including BP, MF, and CC. (B) KEGG enriched analysis of DTDP treatment of DN. (C) GSEA plot showing most enriched GO terms and KEGG pathways in the DN group. (D) PPI network diagram of KEGG pathway and core gene in the top 11, blue represents core gene, and pink represents pathway. (E) Molecular mechanism diagram of core target therapy for DN. BP = biological process, CC = cellular component, DN = diabetes nephropathy, DTDP = Dahuang-Tusizi drug pair, GO = MF = molecular function, KEGG = Kyoto Encyclopedia of Genes and Genomes, MF = molecular function, PPI= protein–protein interaction.

A total of 35 signaling pathways were associated with the effectiveness of DTDP in treating DN. Bubble plots of the top 10 entries were shown in Figure 4A and B. Based on a functional enrichment analysis of the potential targets listed above in the Metascape database, the BP of DTDP in the treatment of DN focused on responses to xenobiotic stimuli, vascular development, leukocyte migration, inflammatory response, extracellular matrix breakdown, regulation of cell-cell adhesion, positive regulation of endothelial cell migration, etc.; MF focuses on serine-type endopeptidase activity, positive and negative regulation of DNA-binding transcription factor binding by RNA polymerase II-specific transcription factors, cytokine receptor activity. The CC sites were mainly located in the extracellular matrix, the membrane of the secretory granules, and on the external side of the plasma membrane. The signaling pathway involved in TNF signaling pathway, MAPK signaling pathway, IL-17 signaling pathway, AGE-RAGE signaling pathway in diabetes complications, etc.

### 3.7. GSEA-based GO and KEGG

Compared with the control group, Figure 4C exhibited GO-BP was mainly enriched in inflammatory reaction, GO-MF was mainly enriched in collagen binding, GO-CC was mainly concentrated in RNA effect complexes, and signaling pathway was mainly enriched in MAPK pathway.

### 3.8. KEGG relationship network construction

A large rectangular area was occupied by genes including JUN, FOS, CCL2, PTGS2, MMP2, CXCR1, CXCR2, EGF, KDR, CD44, and CYP2B6, as depicted in Figure 4D. Intersection of key genes in the pathway with core genes in PPI. We obtained 8 key genes JUN, FOS, CCL2, PTGS2, MMP2, EGF, KDR, CD44, indicating that these genes are involved in various pathways that are integral to the treatment DN process, as shown in Figure 4E.

### 3.9. Molecular docking

**Figure 5.**
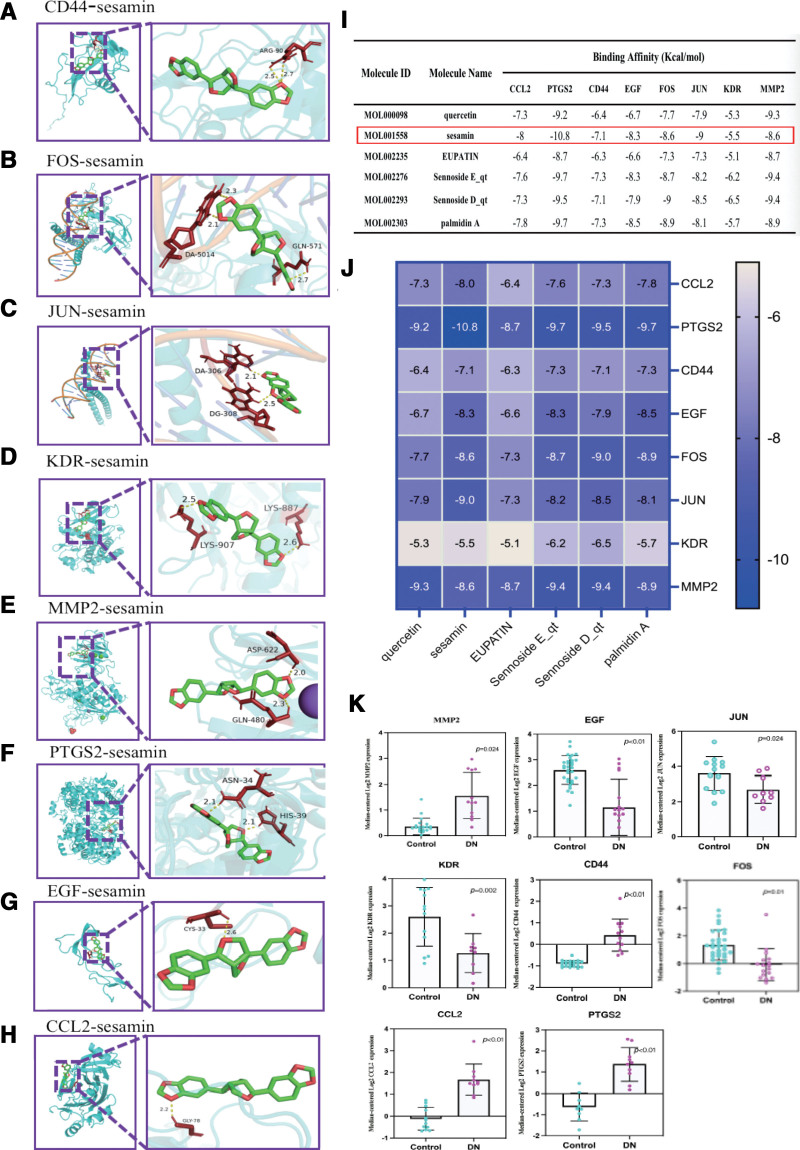
Core protein docking with core components and clinical analysis. (A–H) The molecules and drugs with the lowest docking energies are visualized. The molecular docking of sesamin with CD44, FOS, JUN, KDR, MMP2, PTGS2, EGF, and CCL2 were visualized by Pymol, and the position of hydrogen bonds at the docking sites. (I) The docking energy of 8 core targets and 6 core components in 3-line table. The red box represents the components and core targets with binding energy less than 7.0 kcal mol^−1^. (J) Molecular docking energy heat map of 8 core targets and 6 core components. (K) Association between mRNA expression of hub genes in DN patients and normal controls, *P* < .05 was considered statistically significant. *P < .05, **P < .01, ***P < .001. DN = diabetes nephropathy.

Using molecular docking analysis, we conducted an docking on the significance of 6 vital components in the DTDP and the interplay with 8 essential targets found in DN. By utilizing the PDB database, we identified protein crystals that corresponded with the central target genes. The PDB with the crystal structures of JUN, FOS, MMP2, CCL2, CD44, KDR, PTGS2, EGF are 1jnm, 1s9k, 1qib, 4zk9, 2i83, 6xvk, 5f19, 2kv4. All of the above major targets showed strong binding affinities (<-7.0 kcal mol^-1^) with 6 key active components. The results are shown in Figure 5A–H for the docking diagram of sesamin with 8 proteins, and in Figure 5I and J for the heat map and table. Interestingly, sesamin was found to have the lowest binding energy of all major drugs, showcasing an energy value below −8.0 kcal mol^−1^.

### 3.10. Validations of clinical association between Hub Genes and DN

The results showed that hub genes (JUN, FOS, MMP2, KDR, PTGS2, CD44, EGF, and CCL2) were significantly expressed between DN and normal donors (Fig. 5K). We found that 6 hub genes were significantly higher in DN kidney tissue than in normal kidney samples. While 2 core genes (KDR and EGF) were significantly lower than those in the normal kidney group (*P* < .05). The above results showed the hub genes plays an important role in DN glomerulopathy.

### 3.11. Renal single cell cluster analysis of core genes

**Figure 6.**
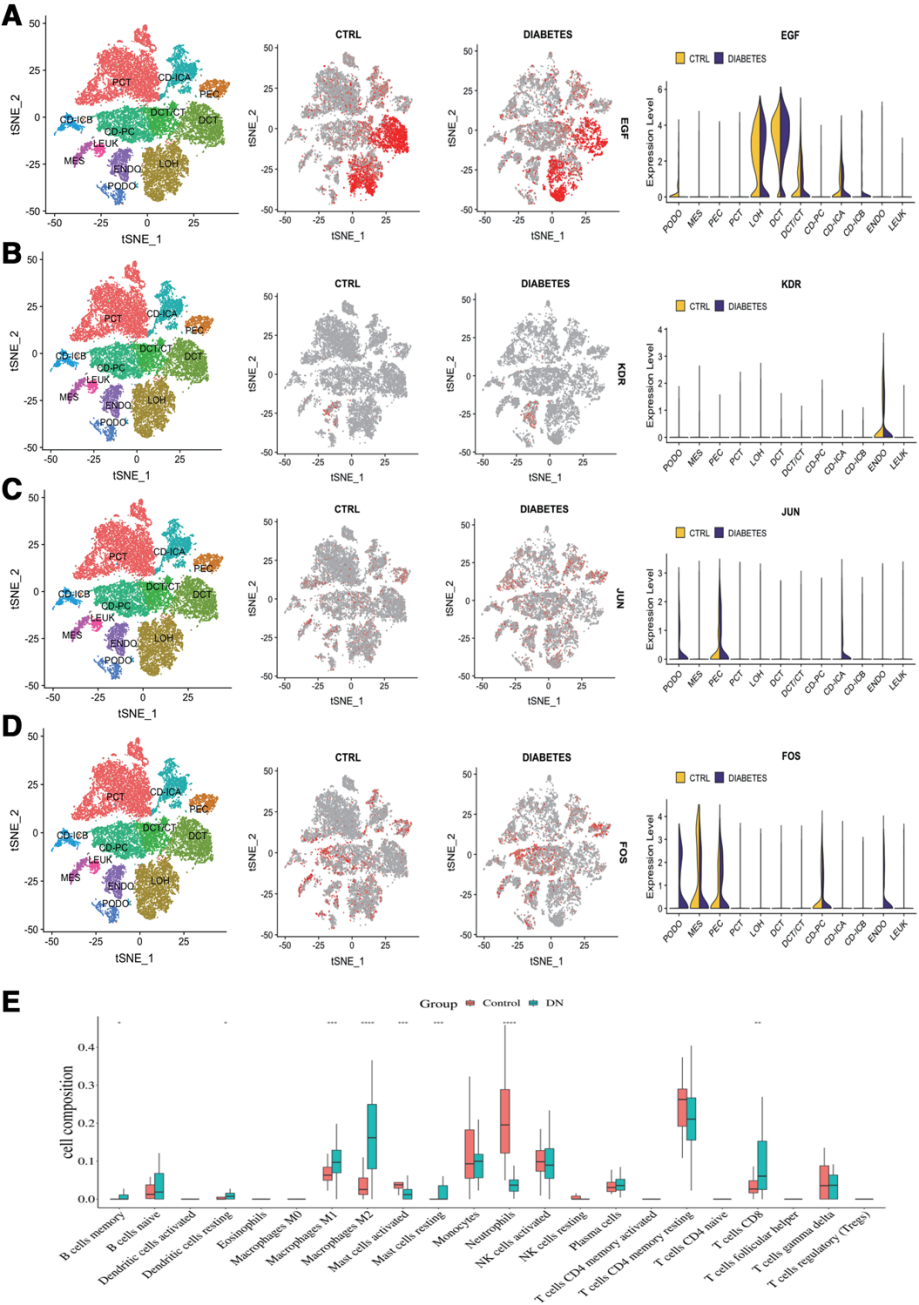
Single cell cluster and ROC analysis of core gene. (A) Expression of EGF in kidney single cell sequencing (CTRL vs DIABETES). (B) Expression of KDR in kidney single cell sequencing (CTRL vs DIABETES). (C) Expression of JUN in kidney single cell sequencing (CTRL vs DIABETES). (D) Expression of FOS in kidney single cell sequencing (CTRL vs DIABETES). (E) ROC curve of core target genes (FOS, JUN, PTGS2, CD44, MMP2, CCL2, EGF, and KDR) for DTDP treatment of DN. DN = diabetes nephropathy, DTDP = Dahuang-Tusizi drug pair.

Single cell cluster analysis can understand the expression of genes in each cell. According to the results, EGF is significantly expressed in the LOH loop and distal convoluted tubules of DN, KDR is expressed in ENDO, and JUN and FOS are widely expressed in all cells of DN. The results are shown in Figure 6A–D.

### 3.12. Analysis of core target gene ROC curve

The area under the ROC curve, known as AUC, is currently considered a standard method for evaluating the accuracy of predictive distribution models. The curve areas of FOS (AUC = 1), JUN (AUC = 0.978), PTGS2 (AUC = 0.927), CD44 (AUC = 0.727), CCL2 (AUC = 0.768), MMP2 (AUC = 0.904), EGF (AUC = 0.884), and KDR (AUC = 0.818) are all above 0.8, further proving the reliability of the above genes as core target genes. The results are shown in Figure 6E.

### 3.13. Evaluation of infiltrating immune cells in DN

**Figure 7.**
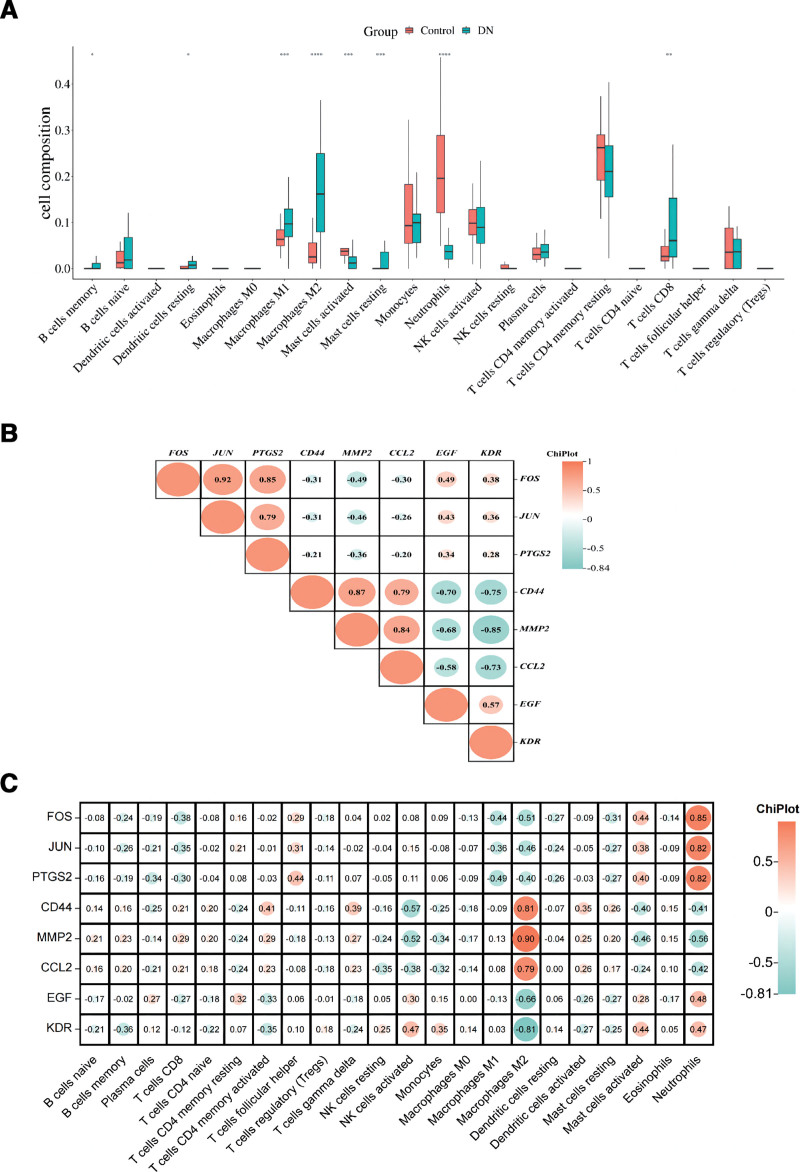
(A) Box plot of the proportion of immune cells in diabetic nephropathy patients and normal samples. The abscissa is the type of immune cell and the ordinate is the proportion of immune cell. Green represents the proportion of immune cells in diabetic nephropathy samples, red represents the proportion of immune cells in normal samples, *P < .05, **P < .01, and ***P < .001. (B) The correlation between core target genes of FOS, JUN, PTGS2, CD44, MMP2, CCL2, EGF and KDR. (C) The correlation between FOS, JUN, PTGS2, CD44, MMP2, CCL2, EGF, KDR core target genes and 22 immune infiltrating cells.

The CIBERSORT algorithm was employed to analyze the GEO data, and boxplots illustrating the proportion of 22 distinct immune cells were generated using the R language. Figure 7A portrays a clear difference, where in the DN samples exhibited noteworthy alterations (*P* < .05) in the abundance of 8 immune cells. Specifically, the percentages of M2 macrophages, CD8 T cells, neutrophils, activated mast cells, and resting mast cells were found to be substantially higher in DN tissue samples compared to the control group. These findings implied that macrophages and other immune factors may have a particularly consequential role in the onset and progression of DN.

### 3.14. Core gene correlation analysis and assessment of immune cell infiltration

The Figure 7B shows that FOS, JUN, and PTGS2 have a strong synergistic effect (Corr > 0.8), while EGF and KDR Respectively with CD4, MMP2, and CCL2 have a negative correlation (Corr > 0.6). MMP2, CCL2, and CD44 have the same effect (Corr > 0.8). In order to understand the molecular mechanisms of immune cell percolation alterations, we need to further explore the relationship between core genes and immune cells. So, we calculated the correlation of 8 core genes with 22 immune cells. The Figure 7C shows that 8 core genes had a significant correlation with M2 macrophages and neutrophils (*P* < .01), among which CD4, MMP2, CCL2 with M2 macrophages had a strong positive correlation (Corr > 0.8), EGF and KDR showed a strong negative correlation with M2 macrophages (Corr < -0.7). FOS, JUN, PTGS2 were strongly positively correlated with neutrophils (Corr > 0.8), CD4, MMP2, CCL2 was negatively correlated with neutrophils (Corr < -0.6).

## 4. Discussion

DN is a serious microvascular complication in DM patients. Several factors are involved in the development of DN, including genetic factors, glomerular hyperfiltration,^[30]^ oxidative stress,^[31]^ accumulation of advanced glycation end-products, activation of protein kinase C,^[32]^ overexpression of transforming growth factor-β, followed by increase of extracellular matrices.^[33]^ In the past few years, substantial evidence has substantiated the notion that anomalies in immune infiltration and inflammatory reaction could play a pivotal role in DN. The occurrence of DN inflammation might involve various types of cells, including renal endothelial cells (mesangial cells, endothelial cells, monocytes, tubular epithelial cells), as well as exogenous cellular elements (macrophages, neutrophils, platelets, lymphocytes, mast cells). These diverse cell types actively contribute to the progression of DN.^[34]^ Furthermore, some pro-inflammatory molecules such as adhesion molecules, chemokines and cytokines play an important role in the development of DN.^[35]^ Therefore, these proinflammatory molecules and inflammatory cells may be current therapeutic targets for DN.

Network pharmacology methods and bioinformatics analysis help to identify putative active ingredients and drug targets. First, in this study, Using GEO2R software to analyze the GSE96804 chip, a total of 654 genes were obtained. There are 314 up-regulated genes and 340 down-regulated genes in DEGs. Subsequently, the Traditional Chinese Medicine Systems Pharmacology database was searched, and a total of 25 active DTDP components were found. At same time, a total of 431 DTDP candidate targets were selected through Swiss Target Prediction. After interleaving the DEGs with the drug target genes, 22 genes were involved in DTDP treatment of DN, with 4 showing upregulation and 18 displaying downregulation, we further explored the PPI core genes and constructed a core gene network. After DC screening, the core genes greatly related to the treatment of DN were finally obtained, including JUN, EGF, CD44, FOS, KDR, CCL2, PTGS2, MMP2 and additional genes formed the core of the network. Among them, CD44, CCL2, PTGS2, MMP2, and JUN were mainly related to the inflammatory response.

According to a research study, it has been demonstrated that JUN (AP-1 transcription factor) represents a heterodimer formed through the combination of FOS protein and JUN protein encoded by the proto-oncogene.^[36]^ It is largely involved in the transcriptional regulation of multiple genes such as cell survival, proliferation and apoptosis.^[37]^ The activation of JUN is critical for mesangial cell proliferation and extracellular matrix production, and mesangial expansion is an important pathological feature of glomerulonephropathy.^[38]^ CCL2, also known as MCP-1, represents a potent type of chemokines, primarily synthesized by tubular epithelial cells and mesangial cells. It is also thought to be an inflammatory factor that promotes DN and is elevated in DN patients.^[39,40]^ CD44 is a type I transmembrane glycoprotein family known as an adhesion molecule. It has a broad distribution in various tissues, including leukocytes, epithelial cells, and endothelial cells. Additionally, CD44 plays a significant role in multiple physiological and pathological processes. These include interactions between cells and between cells and the extracellular matrix, the extravasation of leukocytes, wound healing, cell migration, and the activation of lymphocytes.^[41]^ A study reported that CD44 expression is markedly enhanced in glomeruli and on injured renal tubular epithelial cells and capillary endothelial cells.^[42]^ PTGS2, also known as prostaglandin biosynthesis enzyme, plays a pivotal role in the production of prostaglandins. These prostaglandins have critical implications in processes like inflammation and cell division. Moreover, PTGS2 exhibits various biological actions, including the suppression of apoptosis, facilitation of cell growth, and stimulation of blood vessel formation. These findings imply a significant involvement of PTGS2 in the formation and progression of DN.^[43]^ The EGF is a potent trophic factor produced mainly in kidney tubules, and plays a role in kidney development and in tissue repair.^[44]^ Experimental studies have shown the importance of EGF and KDR signaling in maintaining tubular epithelial cell integrity.^[45]^ In addition, it was demonstrated that matrix metalloproteinases (MMP) levels are implicated in diabetic complications such as retinopathy, cardiomyopathy, peripheral neuropathy and nephropathy.^[46]^ The evidence suggested that MMP-2 is linked to renal hypertrophy and abnormal extra cellular matrix deposition, which are hallmarks of DN.^[47]^ To confirm the robustness of our results compared to external datasets, To examine the expression levels of core genes linked to DN and normal controls, we employed the Nephroseq V5 online database. Statistical significance was defined as a *P* value less than .05. In our study, it was shown that between DN and normal living donors, core genes were significantly expressed. We found that 6 core genes (JUN, FOS, MMP2, PTGS2, CD44, and CCL2) were significantly larger than normal renal samples. Two hub genes (KDR and EGF) were significantly lower than that of normal kidney samples and are statistically significant (*P* < .05). Both the literature reports and the V5 results showed that the expression of JUN, FOS, MMP2, PTGS2, and CD44 were up-regulated in DN. However, KDR and EGF were downregulated in DN.

Simultaneously, we screened 6 key active components from the core of the gene network, such as sesamin, quercetin, EUPATIN, SennosideE_qt, SennosideD_qt and palmidin A. Among them, sesamin, quercetin and EUPATIN have been widely reported in experimental studies for their anti-inflammatory effects. Many studies have shown that Quercetin is a natural flavonoid compound, which has various pharmacological effects such as anti-oxidation, anti-inflammation, anti-cancer, anti-diabetes, and anti-obesity.^[48]^ The administration of Quercetin significantly enhances disease symptoms, renal hypertrophy index, renal histopathology, oxidative stress, and blood glucose levels in a mouse model of DN.^[49]^ Sesamin, a natural lignin compound, and it possesses health beneficial effects, such as anti-aging, anti-cancer, anti-diabetes, anti-inflammatory and antioxidant properties.^[50]^ The incorporation of Sesamin as a natural adjuvant can be effective in improving vascular reactivity and aortic permeability and DN.^[51]^ Eupatin is a polymethoxylated flavonoid that is said to inhibit iNOS and AchE.^[52]^ The studies have shown that eupatin can significantly inhibit the expression of iNOS and COX-2 in macrophages and microglia, as well as the production of nitrite induced by LPS, and has a significant anti-inflammatory effect.^[53]^ In summary, we speculated that the above key ingredients treatment of DN through anti-inflammatory effects.

Meanwhile, In this study, DTDP treatment of DN was subjected to both GO enrichment analysis and KEGG enrichment analysis. The results of GO-BP enrichment analysis revealed that the treatment with DTDP for DN primarily targets inflammatory responses, vascular development, leukocyte migration, breakdown of the extracellular matrix, regulation of cell-cell adhesion, and migration of endothelial cells. Combined with the enrichment analysis of the GSEA, both the DEGs and the GSEA of the GO-BP analysis focus on inflammatory responses. Thus, DTDP plays a role in treating DN through inhibiting inflammatory response. In addition, The GO-MF mainly focuses on serine-type endopeptidase activity, positive and negative regulation of RNA polymerase II-specific DNA-binding transcription factor binding, cytokine receptor activity, oxidoreductase activity, etc. The GO-CC were mainly located in extracellular matrix, secretory granule membrane, and external side of plasma membrane, etc. According to DEGs and GSEA-GO enrichment results, inflammation is closely related to the occurrence and development of DN and it is characterized by excessive deposition of ECM proteins in the mesangium and basement membrane of the glomerulus and in the renal tubulointerstium.^[54]^ Hence, we concluded that DTDP treatment of DN by inhibiting inflammation pathways, reducing extracellular matrix (ECM) deposition. From the enrichment analysis of KEGG, the therapeutic targets of DTDP against DN were mainly enrichment in inflammatory pathways, including MAPK signaling pathway, TNF signaling pathway, IL-17 signaling pathway, advanced glycation end-products-RAGE signaling pathway in diabetic complications, etc. Based on the enrichment analysis of GSEA, the DN samples were mainly associated with the MAPK signaling pathway. Thus, we speculated that DTDP treatment of DN were mainly enrichment in MAPK signaling pathway. As we all know, the MAPK signaling pathway is canonical inflammatory signaling pathway. Many studies have supported that MAPK signaling pathway plays a key role in inhibiting apoptosis, promoting proliferation and inhibiting inflammation in DN rats.^[55,56]^ Therefore, inhibition of the MAPK signaling pathway can attenuate the inflammatory response of DN and protect the kidney.^[57]^

In this study, we used the CIBERSORT algorithm to analyze the immune cell infiltration in the above-gene chip (normal vs DN samples). The results showed that the proportion of M1 macrophages in the DN tissue sample was significantly higher than in the control sample. The macrophages were observed in the glomeruli and interstitium of DN patients, suggesting that immune factors such as macrophages may play a critical role in the development of DN.^[58]^ During the pathogenesis of DN, inflammatory cells proliferate and differentiate, renal tissue cell infiltration increases, and the large amounts of cytokines are secreted to mediate renal tissue injury.^[59]^ It has been reported that macrophage infiltration, one of the hallmarks of DN, is significantly increased in the glomerular tissues of most people with DN.^[60]^ As an essential component of the mononuclear phagocytic system, macrophages play a highly critical role in the development of the body, tissue homeostasis and repair. In general, macrophages have 2 phenotypes, M1 macrophages and M2 macrophages, with distinct functions. M1 macrophages are typical activated cells that secrete so much pro-inflammatory cytokines (IL-1β, iNOS, and TNF-α), Chemokine (MCP-1) and reactive oxygen species promote inflammatory responses that cause additional damage during the pathogenesis of DN.^[35]^ M2 macrophages frequently function as immunosuppressive cells involved in anti-inflammatory responses, tissue remodeling, and tumor progression.^[61]^ Therefore, our focus was on the significant increase of M1 macrophages observed in glomerular samples obtained from patients with DN. Finally, Studies have shown that the docking fraction of the bioactive components to the target structure is less than -7kcal mol^−1^, indicating that their binding affinity is stronger, that is, the smaller the docking fraction, the stronger the binding strength and the lower the binding force.^[62]^ In our study, we screened 6 active ingredients (Sesamin, Quercetin, EUPATIN, SennosideE_qt, SennosideD_qt and palmidin A) and 8 core targets (JUN, FOS, MMP2, KDR, PTGS2, CD44, EGF, CCL2) were verified by molecular docking technology. The result shows that the binding energies of docking are less than −6 kcal·mol^−^1, which means the key active components of DTDP have excellent binding activity with key targets of DN. Among them, Sesamin had the best binding effect on the 7 core target proteins, and the docking scores were all below −8.0 kcal mol^−1^, indicating a significant impact of DTDP treatment on DN. The docking results offer an effective approach to predict the binding mode between DTDP and core DN-associated target proteins, providing valuable guidance for further experimental validation.

However, due to time and funding issues, we only explored a network pharmacological analysis of the potential functional mechanism of action of DTDP on DN. This study has not been verified by experimental data, and our study still has certain limitations. First, additional invitro experiments and clinical validation are needed to support our future research. Second, DEGs were identified by analyzing microarray data published in the GEO database. However, the sample size of DEGs related to DN is small and needs to be strengthened in the future. And, as injury targets for DN, renal innate cells are involved in disease onset and development. Nonetheless, the study did not address how kidney cells and macrophages communicate. Therefore, we will continue to explore the relationship between DTDP and DN in the following future studies. We will investigate the therapeutic effects of DTDP on DN in in vitro experiments and clinical validation. We will investigate the involvement of intrinsic cells of kidney and macrophage cultures in the inflammatory response mechanism. We will compare the epigenetics related database of DN with the active components of DTDP to explore the potential mechanism of DTDP on DNA methylation, histone modification, and noncoding RNA in DN. The molecular mechanism of DTDP in the treatment of DN has been further investigated by means of gene silencing, CO-IP, EMSA, etc.

## 5. Conclusion

**Figure 8.**
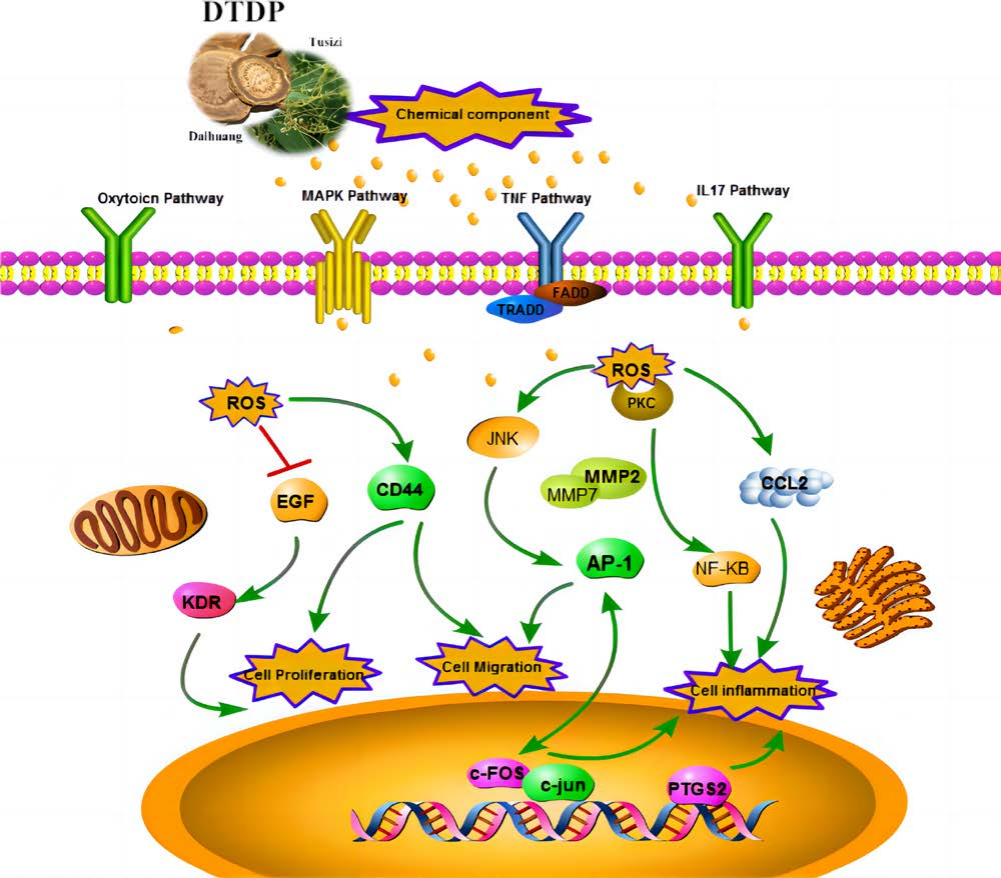
Mechanism of DTDPin the treatment of DN. DL = drug like, DTDP = Dahuang-Tusizi drug pair, OB = oral bioavailability.

In summary, this study combined network pharmacology and bioinformatics to explore the mechanism and molecular targets of DTDP against DN. Our results revealed that DTDP exerts pharmacological effects on DN are “multiple components, multiple targets and multiple pathways.” At the same time, we predict that DTDP may inhibit the inflammatory response by modulating MAPK and TNF inflammatory signaling pathways, reducing macrophage infiltration, thereby reducing glomerular damage and delaying the onset of DN. Thus, DTDP and its key components may be promising drugs for the treatment of DN. And the mechanism of action is shown in Figure 8.

## Author contributions

**Conceptualization:** Wenjing Liu.

**Data curation:** Wenjing Liu.

**Funding acquisition:** Ling Yuan.

**Project administration:** Ling Yuan.

**Software:** Shaozhang Hou, Mengying Che.

**Visualization:** Fandi Men, Duojie Xu.

**Writing – original draft**: Wenjing Liu.

**Writing – review & editing:** Ling Yuan, Yi Nan.

## References

[1] Cho NH Shaw JE Karuranga S. IDF diabetes atlas: global estimates of diabetes prevalence for 2017 and projections for 2045. Diabetes Res Clin Pract. 2018;138:271–. 29496507 10.1016/j.diabres.2018.02.023

[2] Takiyama Y Haneda M. Hypoxia in diabetic kidneys. Biomed Res Int. 2014;2014:837421. 25054148 10.1155/2014/837421 PMC4094876

[3] Nakuluri K Mukhi D Mungamuri SK. Stabilization of hypoxia-inducible factor 1alpha by cobalt chloride impairs podocyte morphology and slit-diaphragm function. J Cell Biochem. 2019;120:7667–78. 30387200 10.1002/jcb.28041

[4] Nakuluri K Mukhi D Nishad R. Hypoxia induces ZEB2 in podocytes: implications in the pathogenesis of proteinuria. J Cell Physiol. 2019;234:6503–18. 30238984 10.1002/jcp.27387

[5] Nakuluri K Nishad R Mukhi D. Cerebral ischemia induces TRPC6 via HIF1alpha/ZEB2 axis in the glomerular podocytes and contributes to proteinuria. Sci Rep. 2019;9:17897. 31784544 10.1038/s41598-019-52872-5 PMC6884642

[6] Guariguata L Whiting DR Hambleton I. Global estimates of diabetes prevalence for 2013 and projections for 2035. Diabetes Res Clin Pract. 2014;103:137–49. 24630390 10.1016/j.diabres.2013.11.002

[7] 2. Classification and diagnosis of diabetes: standards of medical care in Diabetes-2021. Diabetes Care. 2021;44(Suppl 1):S15–33. 34135016 10.2337/dc21-ad09

[8] Gao L Wang XD Niu YY. Molecular targets of Chinese herbs: a clinical study of hepatoma based on network pharmacology. Sci Rep. 2016;6:24944. 27143508 10.1038/srep24944 PMC4855233

[9] Guo MF Dai YJ Gao JR. Uncovering the mechanism of Astragalus membranaceus in the treatment of diabetic nephropathy based on network pharmacology. J Diabetes Res. 2020;2020:5947304. 32215271 10.1155/2020/5947304 PMC7079250

[10] Dong Y Zhao Q Wang Y. Network pharmacology-based investigation of potential targets of astragalus membranaceous-angelica sinensis compound acting on diabetic nephropathy. Sci Rep. 2021;11:19496. 34593896 10.1038/s41598-021-98925-6 PMC8484574

[11] Zhang J Liang R Wang L. Effects and mechanisms of Danshen-Shanzha herb-pair for atherosclerosis treatment using network pharmacology and experimental pharmacology. J Ethnopharmacol. 2019;229:104–14. 30312741 10.1016/j.jep.2018.10.004

[12] Luo Y Xuan C Cheng J. Network pharmacology and in vivo analysis of Dahuang-Huangqi decoction effectiveness in alleviating renal interstitial fibrosis. Evid Based Complement Alternat Med. 2022;2022:4194827. 35774743 10.1155/2022/4194827 PMC9239803

[13] Xiang H Zuo J Guo F. What we already know about rhubarb: a comprehensive review. Chin Med. 2020;15:88. 32863857 10.1186/s13020-020-00370-6 PMC7448319

[14] Hu J Li P Zhang T. Rhubarb combined with trypsin inhibitor for severe acute pancreatitis: a systematic review and meta-analysis. Phytother Res. 2018;32:1450–8. 29672966 10.1002/ptr.6096

[15] Zhang HX Liu MC. Separation procedures for the pharmacologically active components of rhubarb. J Chromatogr B Analyt Technol Biomed Life Sci. 2004;812:175–81. 10.1016/j.jchromb.2004.08.010 15556496

[16] Hu B Zhang H Meng X. Aloe-emodin from rhubarb (Rheum rhabarbarum) inhibits lipopolysaccharide-induced inflammatory responses in RAW2647 macrophages. J Ethnopharmacol. 2014;153:846–53. 24685589 10.1016/j.jep.2014.03.059

[17] Sun W Zhang X Zhang Z. Data fusion of near-infrared and mid-infrared spectra for identification of rhubarb. Spectrochim Acta A Mol Biomol Spectrosc. 2017;171:72–9. 27487576 10.1016/j.saa.2016.07.039

[18] Guo J Chen H Zhao X. Diabetic kidney disease treated with a modified Shenzhuo formula derived from Traditional Chinese Medicine: a case report. J Tradit Chin Med. 2017;37:854–61. 32188197

[19] Ye M Yan Y Guo DA. Characterization of phenolic compounds in the Chinese herbal drug Tu-Si-Zi by liquid chromatography coupled to electrospray ionization mass spectrometry. Rapid Commun Mass Spectrom. 2005;19:1469–84. 15880618 10.1002/rcm.1944

[20] Donnapee S Li J Yang X. Cuscuta chinensis Lam: a systematic review on ethnopharmacology, phytochemistry and pharmacology of an important traditional herbal medicine. J Ethnopharmacol. 2014;157:292–308. 25281912 10.1016/j.jep.2014.09.032

[21] Liao JC Chang WT Lee MS. Antinociceptive and anti-inflammatory activities of Cuscuta chinensis seeds in mice. Am J Chin Med. 2014;42:223–42. 24467546 10.1142/S0192415X14500153

[22] Bao X Wang Z Fang J. Structural features of an immunostimulating and antioxidant acidic polysaccharide from the seeds of Cuscuta chinensis. Planta Med. 2002;68:237–43. 11914961 10.1055/s-2002-23133

[23] Liu J Zhang Y Sheng H. Hyperoside suppresses renal inflammation by regulating macrophage polarization in mice with type 2 diabetes mellitus. Front Immunol. 2021;12:733808. 34925317 10.3389/fimmu.2021.733808 PMC8678409

[24] Li WL Zheng HC Bukuru J. Natural medicines used in the traditional Chinese medical system for therapy of diabetes mellitus. J Ethnopharmacol. 2004;92:1–21. 15099842 10.1016/j.jep.2003.12.031

[25] Fan RH Liu CG Zhang Z. Metabolomics analysis of Semen Cuscutae protection of kidney deficient model rats using ultra high-performance liquid chromatography-quadrupole time-of-flight Mass Spectrometry. J Pharm Biomed Anal. 2022;207:114432. 34715580 10.1016/j.jpba.2021.114432

[26] Wang J Bao B Meng F. To study the mechanism of Cuscuta chinensis Lam and Lycium barbarum L in the treatment of asthenospermia based on network pharmacology. J Ethnopharmacol. 2021;270:113790. 33460759 10.1016/j.jep.2021.113790

[27] Chen L Cao Y Zhang H. Network pharmacology-based strategy for predicting active ingredients and potential targets of Yangxinshi tablet for treating heart failure. J Ethnopharmacol. 2018;219:359–68. 29366769 10.1016/j.jep.2017.12.011

[28] Li S Zhang B. Traditional Chinese medicine network pharmacology: theory, methodology and application. Chin J Nat Med. 2013;11:110–20. 23787177 10.1016/S1875-5364(13)60037-0

[29] Song W Ni S Fu Y. Uncovering the mechanism of Maxing Ganshi Decoction on asthma from a systematic perspective: a network pharmacology study. Sci Rep. 2018;8:17362. 30478434 10.1038/s41598-018-35791-9 PMC6255815

[30] Magee GM Bilous RW Cardwell CR. Is hyperfiltration associated with the future risk of developing diabetic nephropathy? A meta-analysis. Diabetologia. 2009;52:691–7. 19198800 10.1007/s00125-009-1268-0

[31] Agarwal R. Pathogenesis of Diabetic Nephropathy. Chronic Kidney Disease and Type 2 Diabetes. Arlington, VA; 2021:2–7.

[32] Koya D Jirousek MR Lin YW. Characterization of protein kinase C beta isoform activation on the gene expression of transforming growth factor-beta, extracellular matrix components, and prostanoids in the glomeruli of diabetic rats. J Clin Invest. 1997;100:115–26. 9202063 10.1172/JCI119503 PMC508171

[33] Ziyadeh FN Sharma K Ericksen M. Stimulation of collagen gene expression and protein synthesis in murine mesangial cells by high glucose is mediated by autocrine activation of transforming growth factor-beta. J Clin Invest. 1994;93:536–42. 8113392 10.1172/JCI117004 PMC293875

[34] Taniguchi K Xia L Goldberg HJ. Inhibition of Src kinase blocks high glucose-induced EGFR transactivation and collagen synthesis in mesangial cells and prevents diabetic nephropathy in mice. Diabetes. 2013;62:3874–86. 23942551 10.2337/db12-1010 PMC3806624

[35] Navarro-González JF Mora-Fernández C Muros de Fuentes M. Inflammatory molecules and pathways in the pathogenesis of diabetic nephropathy. Nat Rev Nephrol. 2011;7:327–40. 21537349 10.1038/nrneph.2011.51

[36] Karin M. The regulation of AP-1 activity by mitogen-activated protein kinases. Philos Trans R Soc Lond B Biol Sci. 1996;351:127–34. 8650258 10.1098/rstb.1996.0008

[37] Meng Q Xia Y. c-Jun, at the crossroad of the signaling network. Protein Cell. 2011;2:889–98. 22180088 10.1007/s13238-011-1113-3 PMC4875184

[38] Ahn JD Morishita R Kaneda Y. Transcription factor decoy for AP-1 reduces mesangial cell proliferation and extracellular matrix production in vitro and in vivo. Gene Ther. 2004;11:916–23. 14961072 10.1038/sj.gt.3302236

[39] Giunti S Barutta F Perin PC. Targeting the MCP-1/CCR2 system in diabetic kidney disease. Curr Vasc Pharmacol. 2010;8:849–60. 20180766 10.2174/157016110793563816

[40] Park J Ryu DR Li JJ. MCP-1/CCR2 system is involved in high glucose-induced fibronectin and type IV collagen expression in cultured mesangial cells. Am J Physiol Renal Physiol. 2008;295:F749–57. 18579703 10.1152/ajprenal.00547.2007

[41] Rouschop KM Roelofs JJ Claessen N. Protection against renal ischemia reperfusion injury by CD44 disruption. J Am Soc Nephrol. 2005;16:2034–43. 15901765 10.1681/ASN.2005010054

[42] Florquin S Nunziata R Claessen N. CD44 expression in IgA nephropathy. Am J Kidney Dis. 2002;39:407–14. 11840384 10.1053/ajkd.2002.30563

[43] Singh B Kumar A Singh H. Protective effect of vanillic acid against diabetes and diabetic nephropathy by attenuating oxidative stress and upregulation of NF-kappaB, TNF-alpha and COX-2 proteins in rats. Phytother Res. 2022;36:1338–52. 35088468 10.1002/ptr.7392

[44] Humes HD Cieslinski DA Coimbra TM. Epidermal growth factor enhances renal tubule cell regeneration and repair and accelerates the recovery of renal function in postischemic acute renal failure. J Clin Invest. 1989;84:1757–61. 2592559 10.1172/JCI114359 PMC304052

[45] Chen J Chen JK Harris RC. Deletion of the epidermal growth factor receptor in renal proximal tubule epithelial cells delays recovery from acute kidney injury. Kidney Int. 2012;82:45–52. 22418982 10.1038/ki.2012.43 PMC3376190

[46] Abreu BJ de Brito Vieira WH. Metalloproteinase changes in diabetes. Adv Exp Med Biol. 2016;920:185–90. 27535260 10.1007/978-3-319-33943-6_17

[47] Aghadavod E Soleimani A Amirani E. Comparison between biomarkers of kidney injury, inflammation, and oxidative stress in patients with diabetic nephropathy and type 2 diabetes mellitus. Iran J Kidney Dis. 2020;14:31–5. 32156839

[48] Ebrahimpour S Zakeri M Esmaeili A. Crosstalk between obesity, diabetes, and Alzheimer’s disease: introducing quercetin as an effective triple herbal medicine. Ageing Res Rev. 2020;62:101095. 32535272 10.1016/j.arr.2020.101095

[49] Wang C Pan Y Zhang QY. Quercetin and allopurinol ameliorate kidney injury in STZ-treated rats with regulation of renal NLRP3 inflammasome activation and lipid accumulation. PLoS One. 2012;7:e38285. 22701621 10.1371/journal.pone.0038285 PMC3372527

[50] Wu MS Aquino LBB Barbaza MYU. Anti-inflammatory and anticancer properties of bioactive compounds from Sesamum indicum L. – a review. Molecules. 2019;24:4426. 31817084 10.3390/molecules24244426 PMC6943436

[51] Mahmoodi MR Abbasi MM. Therapeutic effectiveness of sesame preparations and its bioactive ingredients in management of cardiometabolic syndrome in diabetes mellitus: a systematic review. Curr Diabetes Rev. 2022. 10.2174/1573399818666220525110925 35619269

[52] Chougouo RD Nguekeu YM Dzoyem JP. Anti-inflammatory and acetylcholinesterase activity of extract, fractions and five compounds isolated from the leaves and twigs of Artemisia annua growing in Cameroon. Springerplus. 2016;5:1525. 27652098 10.1186/s40064-016-3199-9 PMC5017989

[53] Chou CH Hsu KC Lin TE. Anti-Inflammatory and Tau Phosphorylation-inhibitory effects of Eupatin. Molecules. 2020;25:5652. 33266202 10.3390/molecules25235652 PMC7731404

[54] Mason RM Wahab NA. Extracellular matrix metabolism in diabetic nephropathy. J Am Soc Nephrol. 2003;14:1358–73. 12707406 10.1097/01.asn.0000065640.77499.d7

[55] Yong HY Koh MS Moon A. The p38 MAPK inhibitors for the treatment of inflammatory diseases and cancer. Expert Opin Investig Drugs. 2009;18:1893–905. 10.1517/13543780903321490 19852565

[56] Yeung YT Aziz F Guerrero-Castilla A. Signaling pathways in inflammation and anti-inflammatory therapies. Curr Pharm Des. 2018;24:1449–84. 29589535 10.2174/1381612824666180327165604

[57] Song Y Wang X Qin S. Esculin ameliorates cognitive impairment in experimental diabetic nephropathy and induces anti-oxidative stress and anti-inflammatory effects via the MAPK pathway. Mol Med Rep. 2018;17:7395–402. 29568860 10.3892/mmr.2018.8727

[58] Bruchfeld A Wendt M Miller EJ. Macrophage migration inhibitory factor in clinical kidney disease. Front Immunol. 2016;7:8. 26858715 10.3389/fimmu.2016.00008 PMC4726817

[59] Yang X Mou S. Role of immune cells in diabetic kidney disease. Curr Gene Ther. 2017;17:424–33. 29446740 10.2174/1566523218666180214100351

[60] Tang PM Nikolic-Paterson DJ Lan HY. Macrophages: versatile players in renal inflammation and fibrosis. Nat Rev Nephrol. 2019;15:144–58. 30692665 10.1038/s41581-019-0110-2

[61] Mantovani A Sozzani S Locati M. Macrophage polarization: tumor-associated macrophages as a paradigm for polarized M2 mononuclear phagocytes. Trends Immunol. 2002;23:549–55. 12401408 10.1016/s1471-4906(02)02302-5

[62] Hsin KY Ghosh S Kitano H. Combining machine learning systems and multiple docking simulation packages to improve docking prediction reliability for network pharmacology. PLoS One. 2013;8:e83922. 24391846 10.1371/journal.pone.0083922 PMC3877102

